# Enhanced Acoustic Properties of a Novel Prepacked Aggregates Concrete Reinforced with Waste Polypropylene Fibers

**DOI:** 10.3390/ma15031173

**Published:** 2022-02-03

**Authors:** Rayed Alyousef, Hossein Mohammadhosseini, Ahmed Abdel Khalek Ebid, Hisham Alabduljabbar, Shek Poi Ngian, Ghasan Fahim Huseien, Abdeliazim Mustafa Mohamed

**Affiliations:** 1 Department of Civil Engineering, College of Engineering, Prince Sattam bin Abdulaziz University, Al-Kharj 11942, Saudi Arabia; h.alabduljabbar@psau.edu.sa (H.A.); eng.gassan@yahoo.com (G.F.H.); a.bilal@psau.edu.sa (A.M.M.); 2 Institute for Smart Infrastructure and Innovative Construction (ISIIC), School of Civil Engineering, Universiti Teknologi Malaysia (UTM), Skudai 81310, Malaysia; mhossein@utm.my (H.M.); shekpoingian@utm.my (S.P.N.); 3 Faculty of Engineering and Technology, Future University in Egypt, New Cairo 11845, Egypt; Ahmed.AbdelKhaleq@fue.edu.eg; 4 Department of the Build Environment, School of Design and Environment, National University of Singapore, Singapore 117566, Singapore; 5 Building and Construction Technology Department, Bayan University, Khartoum 11115, Sudan

**Keywords:** acoustic behavior, prepacked aggregates fiber-reinforced concrete, waste polypropylene fiber, sound absorption coefficient

## Abstract

This research aimed to investigate the performance of prepacked aggregates fiber-reinforced concrete (PAFRC) with adequate acoustic characteristics for various applications. PAFRC is a newly developed concrete made by arranging and packing aggregates and short fibers in predetermined formworks, then inserting a grout mixture into the voids amongst the aggregate particles using a pump or gravity mechanism. After a one-year curing period, the effects of utilizing waste polypropylene (PP) fibers on the strength and acoustic characteristics of PAFRC mixes were examined. Compressive and tensile strengths, ultrasonic pulse velocity (UPV), sound absorption, and transmission loss were investigated on plain concrete and PAFRC mixtures comprising 0–1% PP fibers. The results revealed that the use of PP fibers slightly decreased the compressive strength and UPV of PAFRC mixes. The inclusion of waste PP fibers also significantly increased the tensile strength and sound insulation coefficient of PAFRC mixes, especially at higher fiber dosages. In the medium-to-high frequency ranges, more than 60% acoustic absorption coefficient was observed, indicating that PAFRC specimens have good sound insulation properties.

## 1. Introduction

In a modern lifestyle, noise is becoming a significant source of concern due to the adverse effects on many people’s lives in urban and rural regions. Noise pollution from various sources, such as cars, planes, power plants, and industry, is inconvenient and harmful to human health. As a result of these issues, considerable advancements in sound absorption materials, particularly in the construction sector, have occurred. There are several empirically verified constants for acoustic performance for homogeneous and isotropic materials, including absorption and reflection coefficients as well as acoustic impedance [1,2,3]. Prepacked or preplaced aggregate concrete (PAC) is a type of concrete made by placing aggregates in the premeditated formworks, then filling the voids and cavities between the aggregated particles with a flowable cement-based mixture grout [4]. In contrast to typical concrete, the packing method permits more particles to compress into formworks and interconnect with one another [5]. This interconnecting behavior increases aggregate contact and minimizes void volume, resulting in a well-distribution of stresses under loading and, subsequently, improved concrete component performance [6].

Grouting is done in one of two ways in the manufacturing of PAC—by gravity or by high-pressure injection pumps. Accordingly, the application and minimum aggregate size employed are essential factors to consider when selecting a proper technique for grouting amongst aggregates [7]. As the settlement of coarse aggregates in PAC is congested with the small size of cavities in uses where smaller aggregates are used, and the depth of molds is greater than 30 cm, the grout injection technique by a controlled-pressure pump is preferred. In contrast, the gravity method of grouting is selected in the formworks with depths of up to 30 cm and larger sizes of aggregates [8,9]. PAC is preferable when restoring existing concrete components and various infrastructure projects that call for a specialized approach. It is also possible to utilize pre-mixed concrete such as PAC in places where conventional concrete compromises the formwork’s integrity, including pipelines, abutments, bridges, and tunnel opening [10,11].

Concrete comes in various forms, each with its characteristics that make it the most widely used construction material, including low cost, ease of development, and high compressive strength. However, when subjected to tensile loads, it performs poorly and exhibits brittle behavior. Furthermore, the performance of concrete components employed as sound and heat resistance materials is influenced by environmental circumstances, mechanical properties, and the components utilized in concrete [12]. Thus, unique and modified materials with increased ductility are required for this type of use [13,14]. Because of PAC’s exclusive process and high performance, it can be employed as sound-isolating concrete panels.

Residents’ safety and well-being have become increasingly critical as the usage of structural concrete has increased. Concrete used as structural members must be adequately insulated against sound heat [15]. Consequently, a novel sort of concrete with more excellent noise pollution resistance is required to improve these specific qualities of concrete and achieve the required performance. Incorporating short fibers with a maximum length of 30 mm into PAC mixtures could be a viable alternative for achieving the desired characteristics in concrete mixes [16]. By releasing energy during pull-out, fibers in the path of a spreading crack bridge the fracture opening and impede further crack propagation. Polypropylene in particular and other fibers are generally used in concrete components exposed to high dynamic loads. These fibers significantly improve the tensile and flexural strength and sound isolation in concrete [17,18]. The current research offered a new PAC reinforced with short fibers with sufficient sound resistance and noise reduction capabilities. Prepacked aggregates fiber-reinforced concrete (PAFRC) is a unique kind of concrete made of coarse aggregate particles with various shapes and sizes, discontinuous PP fiber with a maximum length of 30 mm, and a flowable grout mixture.

Furthermore, with the advancement of non-destructive testing (NDT) technology and acoustic emission (AE) technique in terms of damage monitoring, recognition, and evaluation of concrete sound isolation property, the effects of fibers and their roles in a concrete matrix and the design of engineering structures have been investigated [19,20,21,22]. To date, much research on the acoustic characteristics of conventional plain and fiber-reinforced concretes has been undertaken. To the best of the authors’ knowledge, no experimental studies in the scientific literature report on the acoustic properties of plain PAC or PAFRC. However, several studies on the sound reduction properties of concrete with the addition of short steel and PP fibers, waste crumb tire, waste shells as partial aggregates have been reported in this regard [23,24,25,26].

Khankhaje et al. [26] pointed out that conventional plain concrete has sound absorption coefficients in the range of 0.03–0.05. In contrast, concrete mixtures with a high porosity volume have an absorption coefficient ranging from 0.1 for poorly accomplished combinations to approximately 1 for mixtures with a higher volume of pores. This is because the material’s cavities absorbed the sound energy over inner friction. Moreover, the noise reduction values of pervious and standard asphalt pavements were compared by Meiarashi et al. [27]. They discovered that pervious asphalt reduced noise by 2–5 dB and concluded that keeping the pavement’s porosity at about 20% or greater was critical for noise reduction. Kim and Lee [28] also examined the influences of different cement flow and various aggregate types on porous concrete’s sound absorption and mechanical characteristics. They discovered that specimens containing smaller aggregates obtained superior sound absorption than the control samples. This was because the specimens’ total void ratio increased by using a smaller size of aggregates, resulting in better sound absorption properties.

Karimipour et al. [29] also looked into the impact of PP fibers on the acoustic properties of concrete with coal coarse particles. They found that adding coarse coal aggregates and PP fibers to concrete significantly improved sound insulation and noise reduction properties. Their findings revealed that with the addition of 2% PP fibers, the sound absorption coefficient of 67% was noted at the frequency of 2000 Hz. The addition of steel fibers up to 0.5% lowered the sound isolating characteristics of concrete, according to Vidya Sagar et al. [30]. They discovered that as the amount of steel fiber in the mix increased and the AE parameters raised, which indicates more AE is being released. They also reported that the peak loads for specimens with 0.3 % fibers were extremely high, and the AE parameters reached a high level.

The effect of adding waste PP fibers on PAFRC’s acoustic characteristics was investigated to establish the possibility of adopting fiber enrichment as a noise reduction technique. As PP fibers are a waste product from the carpet industry, they are quite low priced, and their use has the added advantage of reducing environmental pollution. This research aims to determine the optimal amount of waste PP fibers necessary to enhance sound absorption in PAFRC concrete as a function of fiber content and grouting method across the frequency range of 0–4000 Hz.

## 2. Experimental Program

### 2.1. Materials and Proportions

**Figure 1:**
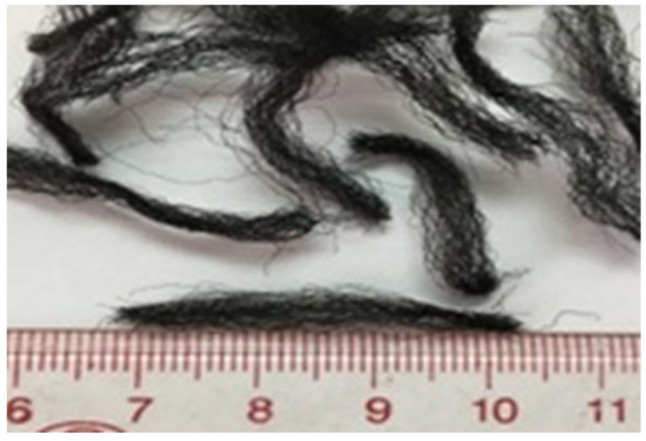
Size and shape of PP fibers used.

This study used ordinary Portland cement (OPC) to produce the high flowable grout mixture. Palm oil fuel ash (POFA) was also used as partial cement at the replacement level of 25%. POFA ashes were analyzed in accordance with ASTM C618-05 and categorized as class C ash. The properties and compositions of OPC and POFA are tabulated in Table 1. To obtain a high-quality and flowable grout mixture, blended cement particles were mixed with natural river sand with a maximum size of 4.75 mm. According to ACI 304.1R-97, which specifies the properties of coarse aggregates for PAC, the crushed granite aggregate particles of sizes between 20 to 40 mm with water absorption of 0.5% and specific gravity of 2.7 g/cm^3^ were used. The fibers used in this research are multi-filament PP fibers collected as waste fibers from carpet industries. Following the pre-mixes and the maximum aggregates, the fibers were cut at 30 mm and used in the main experiments. Figure 1 and Table 2 show the waste PP fibers’ appearance and properties used in this study. Furthermore, 12 concrete mixes in two groups based on the grouting technique were made: pumping (P) and gravity (G). Of these 12 batches, two mixtures—G-0 and P-0—were produced as controls for both pumping and gravity processes without fibers. Fibers were then added to the mixes at doses of 0.2, 0.4, 0.6, 0.8, and 1.0% for the pumping method, P-1 to P-5, and G-1 to G-5 for the gravity method. The mixed proportions of PAFRC mixtures are given in Table 3. 

### 2.2. Sample Preparation and Test Methods

**Figure 2:**
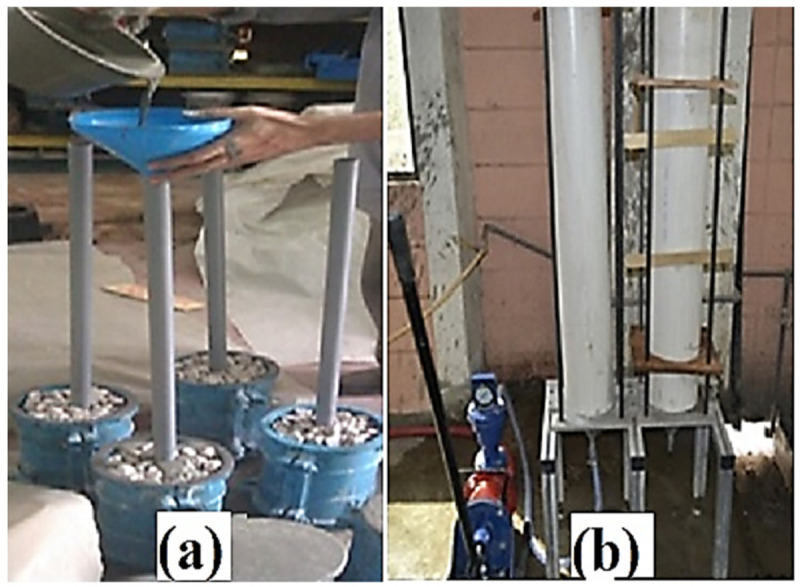
(**a**) Gravity and (**b**) Pumping methods of grouting in PAFRC.

**Figure 3:**
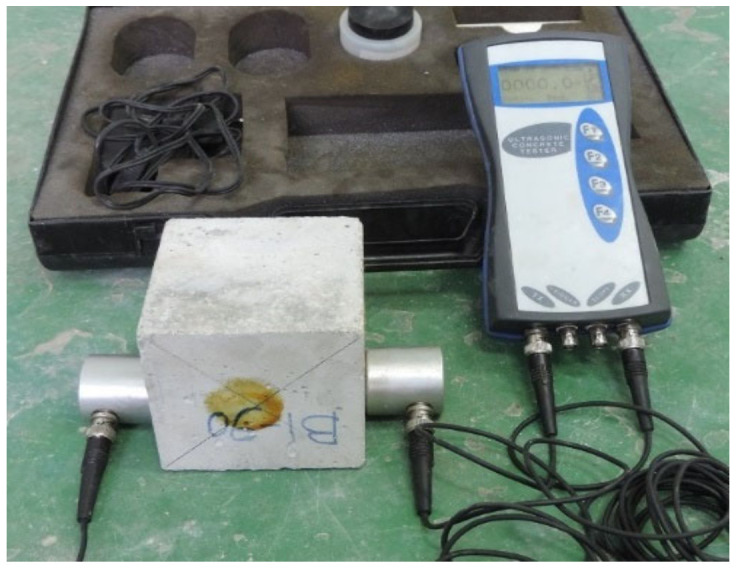
The ultrasonic pulse velocity test device.

PAFRC samples, unlike regular concrete, were produced in two stages. The necessary proportion of aggregates was mixed with the required dosage of PP fibers in the pan mixture as a dry mix. The aggregates and fibers were then positioned in the premeditated molds. After adequately placing aggregates, the grout mixture was injected into the molds, either by gravity or pump. This study used 5-mm diameter PVC pipes for the grouting procedure under gravity force and located at the center of cylindrical molds, as shown in Figure 2a. In the pumping method, UPVC pipes with diameters of 100 mm and 150 mm were used as molds, and the grout was injected between aggregates using a control pressure pump. As revealed in Figure 2b, an appropriate formwork of plywood was built to avoid mold throughout the pumping procedure. To avoid overflowing the grout from the top of the molds, a plywood cap was also positioned on the top surface of the pipes. After the grouting was finished, the specimens were covered for 24 h, the molds were opened, and the samples were immersed in water and cured for 365 days. The cylindrical specimens of a size of 100 mm × 200 mm and 150 mm × 300 mm were made for various tests. ASTM C39M-18 and ASTM C496-17 requirements were followed for the compressive and tensile strength testing, respectively. In addition, as shown in Figure 3, the samples were examined for a non-destructive test of ultrasonic pulse velocity (UPV) in line with ASTM C597-09. For each test, three samples were tested after one year of curing, and the average values were noted as experimental results of this study. 

**Figure 4:**
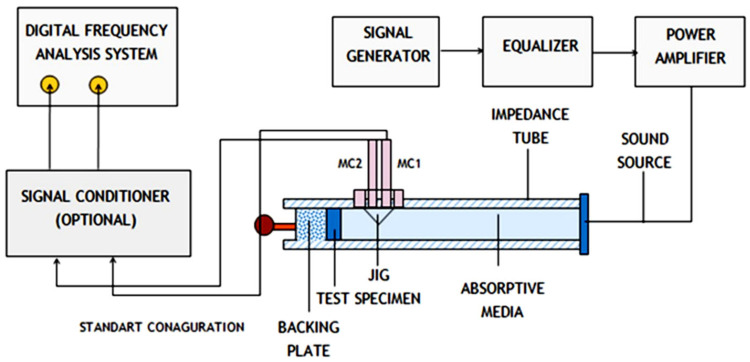
Configuration of impedance tube and experimental setup for acoustic transmission according to ASTM E1050.

Furthermore, the cylindrical samples with sizes of 99 mm × 100 mm and 28 mm × 100 mm were utilized to assess the sound absorption coefficient, noise reduction, and transmission loss analyses of the PAFRC specimens. To this aim, an impedance tube test, as revealed in Figure 4 was utilized. The sound absorption measurement consisted of an impedance tube sealed at both ends, with a source sound speaker generating the standing wave at one end and the test material set against a solid non-transmitting wall. Two microphones positioned within the tube’s wall measured the sound pressure levels. The sound transmission measurement is based on the same principles, except that the test sample is put in the sound field and allowed to flow through. Two microphones were placed before and after the sample, allowing the transmission loss to be calculated. A signal generator, power amplifier, and spectrum analyzer were all part of the test equipment. Generally, the frequencies of the impedance tube are in the range of 50–20,000 Hz, a frequency response of ≤±0.3 dB, and an amplitude range of 30 dB was tested. However, in this study, the frequency range of the determination was 50–4000 Hz, limited in the high-frequency side because of the fixed diameter of the impedance tube and the uncertainty involved in obtaining small phase differences at low frequencies. The gap is sealed with grease once the sample is inserted into the setup. This study applied low-frequency ranges of 50–1500 Hz and high-frequency ranges of 1500–4000 Hz, according to ASTM E1050-19 specifications. The noise reduction coefficient (*NRC*) is a single value that can be used to calculate a material’s ability to absorb sound, as shown in Equation (1) [26]. The sound transmission loss coefficient (TLC) and the sound transmission class (STC) were also analyzed using ASTM E413-16, as PAFRC can be employed as structural or non-structural wall panels.

*NRC* = (*α*_250_ + *α*_500_ + *α*_1000_ + *α*_2000_)/4 (1)

where, *α* is the sound absorption coefficient at various frequencies.

## 3. Results and Discussion

### 3.1. Mechanical Properties 

**Figure 5:**
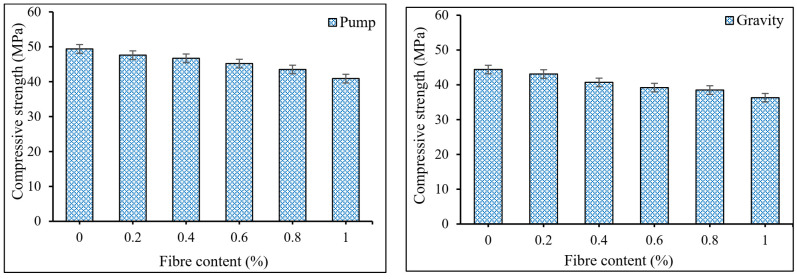
Influences of PP fibers on the compressive strength of PAFRC mixtures.

Concrete’s compressive and tensile strengths are essential for structural designs. Figure 5 illustrates the effect of PP fiber content on the compressive strength of PAFRC mixtures. It can be seen that the attained compressive strengths of PAFRC samples reinforced with 0–1% PP fibers ranged from 36.3 MPa to 49.4 Mpa for gravity and pumping groups. With the addition of PP fibers, the compressive strength decreased slightly. In the pumping method, the mixes containing 0.2, 0.4, 0.6, 0.8, and 1% PP fibers had strength values of 47.6, 46.7, 45.2, 43.5, and 40.9 MPa; correspondingly, all are lower than the 49.4 MPa obtained for the control plain mix. Furthermore, at similar fiber dosages, compressive strength values of 43.1, 40.7, 39.2, 38.5, and 36.3 MPa were achieved in the gravity mixes, which are lower than the plain gravity mix’s 44.4 MPa. It is worth noting that the measured compressive strength values for both gravity and pumping techniques PAFRC mixtures containing PP fibers are over 30 MPa, indicating that they could be used as structural components in various applications [31]. However, the high fiber content in mixes slightly decreased the compressive strength of PAFRC mixtures. The lower strength of the reinforced mixtures might be attributed to excessive dosages of extra fibers, which disrupt the normal movement of grout mixture—mainly in the gravity method mixes—and therefore, improper supply of grout amongst the aggregates and fibers results in lower strength in PAFRC mixes [12]. 

Moreover, both the pumping and gravity mixes showed a similar compressive strength trend. The uniform and proper distribution of grout mixture through a pump with controlled pressure results in a high-quality matrix and, consequently, develops higher strength values than the gravity technique. In addition, the existence of POFA as partial cement in the grout mixture resulted in better matrix performance. It could be due to the pozzolanic behavior of POFA ashes, which contain a high amount of reactive SiO_2_. These SiO_2_ components react with hydration products from OPC and produce supplementary C-S-H gels at ultimate ages and, thus, enhance the strength performance of PAFRC mixes [17].

**Figure 6:**
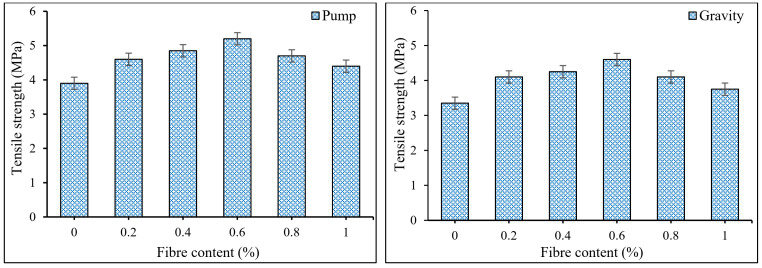
Influences of PP fibers on the tensile strength of PAFRC mixtures.

Figure 6 shows the influence of PP fiber on the tensile strength of PAFRC mixes after one year of water curing for various fiber volume fractions. The PP fiber has a considerable impact on the splitting tensile strength and ductility of concrete. Incorporating 0.2, 0.4, 0.6, 0.8, and 1% PP fibers in the pumping group PAFRC mixes resulted in tensile strength values of 4.6, 4.85, 5.2, 4.7, and 4.4 MPa, correspondingly, and all are higher than the plain mix’s 3.9 MPa. With the gravity method, a similar trend of increasing tensile strength of PAFRC mixtures was seen, although at a slower rate. The tensile strength values for the fiber dosages of 0.2, 0.4, 0.6, 0.8, and 1% were 4.1, 4.3, 4.6, 4.2, and 3.75 MPa, respectively, which are all greater than the plan gravity mix’s 3.35 MPa. Pumping technique PAFRC mixes have a more excellent tensile strength value than gravity method PAFRC mixes, indicating that well-distributed grout maximizes tensile strength increase. The enhanced tensile strength of PAFRC mixtures is attributed to the strong bond between the fibers and aggregates by high-quality grout mixture and the linking action of fibers preventing the formation and spread of microcracks which, consequently, increased the splitting tensile strength of fiber-reinforced mixes as associated to those of plain mixtures [32]. Accordingly, increasing the amount of PP fiber in PAFRC specimens may improve the structural deformation capacity.

**Figure 7:**
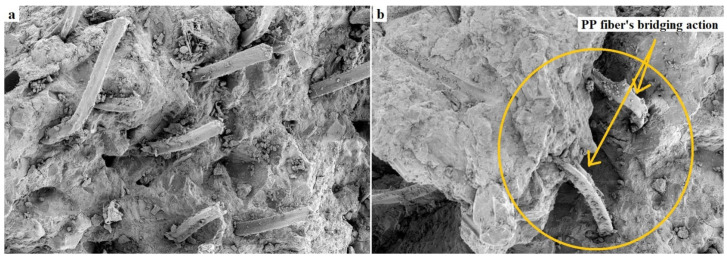
(**a**) Distribution of PP fibers in the matrix and (**b**) the fiber’s bridging action.

The interaction of fiber effects such as crack propagation limitation and air void expansion could alter the compressive and tensile strengths of PAFRC mixtures. The addition of fibers could inhibit further cracks development and enhance tensile strength and ductility. The minimum and maximum enhancements in tensile strength of PAFRC mixtures with PP fibers, as shown in Figure 6, are around 12.8 % and 37% for the pumping method and 11.9 % and 33.3 % for the gravity method, respectively, as compared to plain mixtures. The fiber bridging effect transfers tensile stress across the cracks, enhancing the tensile strength of PAFRC mixtures. Due to the excellent bonding and even spreading of the PP fiber into the matrix, the PAFRC samples exhibited ductile behavior during the early crack generation, as illustrated in Figure 7. The debonding and bridging action of the PP fibers from their surrounding matrix could explain the improved ductility performance of PAFRC specimens. However, further increases in fiber content beyond 0.6% resulted in lower tensile strength. This phenomenon could be due to the increase in the matrix porosity with low flowability of grout at a high-volume fraction as well as the non-uniform fiber distribution of fibers [16]. The inter-particle friction between fibers and aggregates also affects the orientation and distribution of the fibers and, therefore, the tensile strength of the concrete mixtures.

### 3.2. Ultrasonic Pulse Velocity

Non-destructive tests such as ultrasonic pulse velocity have been extensively used to evaluate long-term performance and monitor inner destruction progression in cement-based materials. An ultrasonic measurement on concrete should yield the speed and attenuation of longitudinal waves. The mechanical and durability properties, the microstructure of the matrix, the quality of bonds between components, and dispersed micro-damage all impact these ultrasonic parameters. Consequently, the observed ultrasonic waves speed and attenuation data can be used to evaluate the characteristics of concrete components during their service life. There have been few theoretical investigations into demonstrating ultrasonic waves propagation in the concrete matrix; ultrasonic research on concrete has traditionally been exclusively investigational, and hence, assessments based on ultrasonic testing have frequently been qualitative and data-driven [17].

**Figure 8:**
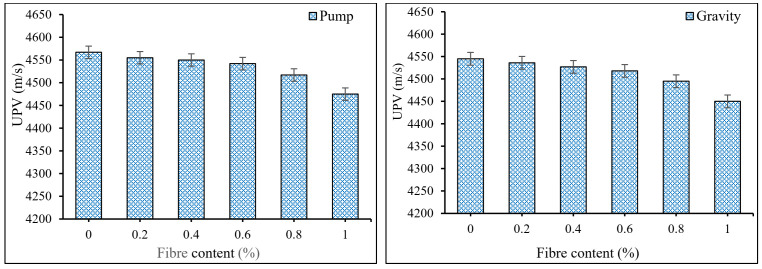
The variation in UPV values of PAFRC mixtures with different fiber contents.

Figure 8 demonstrates the outcomes of the UPV test for PAFRC specimens after one year of curing. It was discovered that the inclusion of PP fibers slightly lowered the UPV values marginally. Plain pumping and gravity plain PAC mixes corresponded with UPV values of 4567 m/s and 4545 m/s. Furthermore, pumping technique PAFRC mixtures containing 0.2, 0.4, 0.6, 0.8, and 1% PP fibers had UPV values of 4555, 4550, 4542, 4517, and 4475 m/s, respectively. The UPV values for the gravity method mixtures reinforced with similar fiber doses were 4536, 4527, 4518, 4495, and 4450 m/s, respectively. Therefore, according to the obtained results, all PAFRC specimens in this study are categorized as good grade concrete, as the attained UPV values for all mixtures were greater than 4400 m/s [33]. The decrease in UPV values, on the other hand, might be related to the existence of PP fibers at high dosages and increases the volume of voids in the matrix [17]. It was found that in pumping technique samples, the volume of voids reduced due to the proper circulation of grout between the aggregates, resulting in higher UPV values.

**Figure 9:**
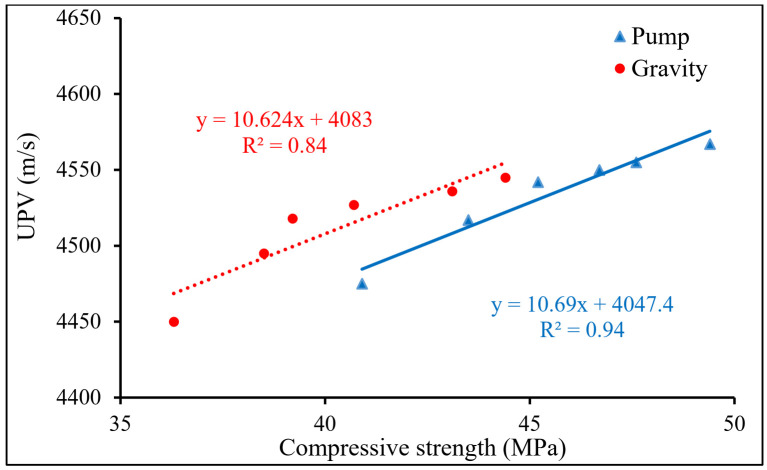
The correlations between UPV and compressive strength of PAFRC mixtures.

The results demonstrated that the compressive strength and the UPV values followed the same pattern by adding PP fiber; hence, the results were correlated, as shown in Figure 9. The outcomes discovered an excellent correlation between the reported compressive strength and the UPV data. This relationship was studied using a linear regression approach. The coefficient of determination (R^2^) for this connection was 0.94 and 0.84 for the pumping and gravity groups, respectively, indicating a high level of confidence in the correlation amongst these two properties of PAFRC mixtures comprising PP fibers at various dosages. This correlation could be beneficial to monitor and predict the strength properties of concrete structures during their service life [34].

### 3.3. Acoustic Properties

Acoustic is the science and technology of sound in and around buildings. It investigates sound reflection, absorption, and insulation phenomena and uses what it learns for noise management and to design rooms with unique acoustic properties, such as concert halls and theaters. Sound insulation is perhaps the most common criterion to be met by practitioners. It is worth noting that many construction projects aim to improve their systems’ acoustic characteristics to the levels attained by concrete. In the past, the sound-insulating capabilities of walls were taken for granted. The sound absorption coefficient of a material, which is the incident sound energy that is absorbed by the medium and not reflected, determines how well it absorbs sound. The absorption coefficient of typical concrete ranges from 0.05 to 0.10. The sound absorption coefficient indicates how well concrete can inhibit sound transmission; a high number shows that concrete can effectively insulate sound [26]. A standing wave tube was used to investigate the sound absorption coefficient, noise reduction, and transmission loss. 

**Figure 10:**
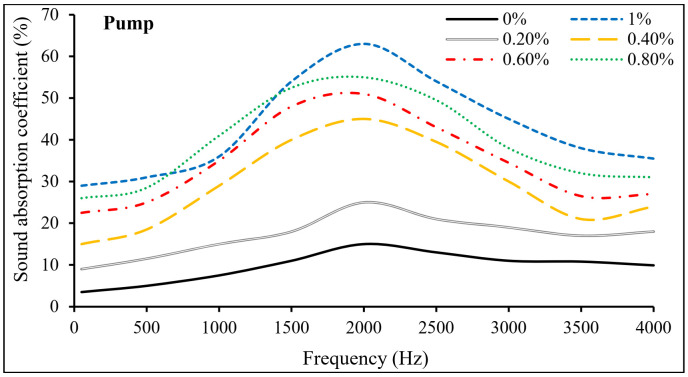

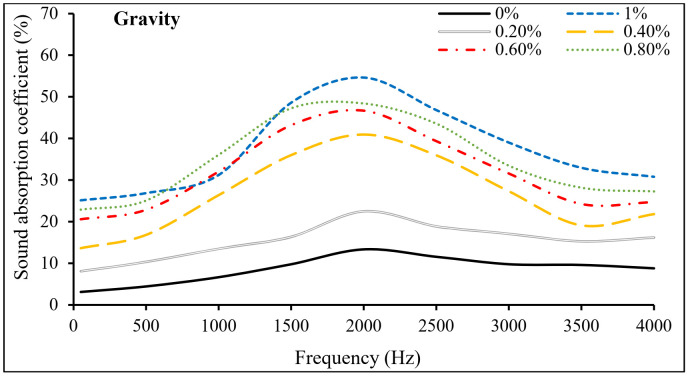
The sound absorption coefficient of PAFRC mixtures.

As shown in Figure 10, PP fibers were beneficial in increasing the sound absorption coefficient and improving the sound insulation of PAFRC mixtures. It can be seen that reinforced specimens could absorb more sound over the entire frequency range than plain mixes. At a frequency of 2000 Hz, the sound absorption coefficient of the PAFRC mixture comprising 1% PP fiber was 60%, which is significantly better than the 15% obtained for the control plain mix. Similarly, in the gravity method PAFRC including 1% fibers, this value was reported as 54.6%, greater than the plain mix’s 13.4%. PP fibers enhanced the sound insulation and substantially improved the sound absorption coefficient, which was especially noticeable in the pumping technique specimens.

Figure 10 shows the variation of absorption coefficients as a function of frequency, demonstrating that varied fiber doses are characterized by minor absorbance below 1000 Hz, where the absorption coefficients are less than 30%. However, over the 1500-hertz frequency band, there was a significant increase in absorption levels, with a high of over 50% for frequencies between 1500 and 2500 Hz. Because the maximum is restricted inside a narrow frequency interval, these absorption maxima can be defined as selective for a specific frequency band. Several types of concrete have been shown to have reduced absorption at low-frequency ranges; nevertheless, only extremely lightweight concretes, such as autoclaved aerated concrete with density values about 400–800 kg/m^3^, show substantial absorption at these low frequencies. When selective adsorption is required without affecting the remainder of the audible spectrum, the absorption between 1500 and 2500 Hz, which is thought to be the zone of highest frequency sensitivity for human hearing, is quite useful. One of these applications would be the absorption of road transportation in densely populated areas with speed limits of 40–50 km/h because the emission spectra for these infrastructures are characterized by significant tread noise in the medium and high-frequency bands [26].

**Figure 11:**
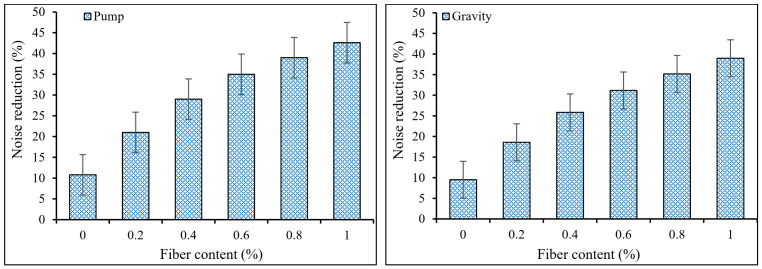
Noise reduction coefficients of PAFRC mixtures.

The noise reduction coefficient evaluated the sound absorption performance under various frequency circumstances. As revealed in Figure 11, the use of PP fibers reduced noise and improved concrete’s sound insulation capacity. Sound absorption was better in the PAFRC mixtures with PP fiber than in the plain mixes. The *NRC*s for the mixtures reinforced with 1% PP fibers, for example, were 42.6% and 38.95% in the pumping and gravity procedures, respectively, which indicates the higher sound absorption properties of the reinforced specimens as compared to those of plain mixes with *NRC* values of 10.8% and 9.5%. The reinforced PAFRC mixes’ superior sound absorption ability could be due to the matrix’s linked fibers reflecting sound inside the voids [27,29]. The continuous dispersion and bridging actions of PP fibers are well seen in Figure 7. These fibers act as a bridge between the concrete constituents and prevent crack progression.

**Figure 12:**
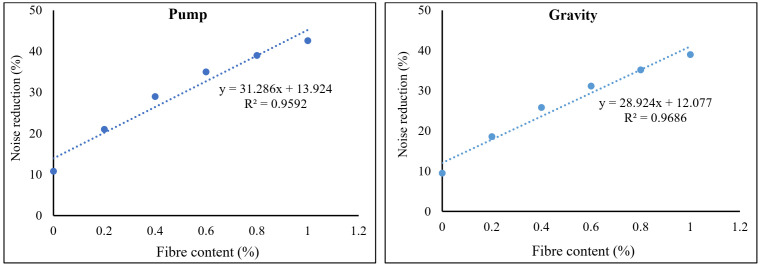
Correlations between noise reduction and fiber content of PAFRC mixes.

Furthermore, the inclusion of PP fibers shows that the concrete matrix and fibers are sufficiently bonded. The bundle of PP fibers provides good resistance against sound pollutions, resulting in increased sound absorption rates. Consequently, the sound began to vibrate and convert to heat [27]. Furthermore, because PAFRC mixtures incorporate blending aggregates of various shapes and sizes, this blended aggregate system in most cases resulted in enhanced sound absorption. Pastor et al. [35] found that adding glass fibers to concrete increases the sound absorption coefficient. Furthermore, Karimipour et al. [29] claimed that adding up to 2% PP fibers to concrete mixtures containing untreated coal coarse aggregates at varied replacement amounts increased the sound absorption coefficient by roughly 45%. Empirical relationships were used to correlate the obtained acoustic properties in terms of noise reduction and fiber volume fractions. These relationships were discovered by data analysis using linear regression. For both gravity and pumping method PAFRC specimens, Figure 12 demonstrates the empirical equations generated between noise reduction and fiber content. According to the findings, the fiber content and acoustic qualities of the PAFRC mixes had a linear connection, with coefficients of determination (R^2^) values more than 0.95. According to the findings, increased fiber doses improved the acoustic characteristics of the PAFRC blends. This result is consistent with Karimipour et al.’s [29] findings. Furthermore, compared to gravity technique specimens, there was a stronger association between noise reduction and fiber concentration in pumping method specimens.

Numerous noise sources depend on the application, and they all produce sound waves directly in the air. Airborne sound and structure carried sound is produced in residential buildings by above neighbors strolling across the floor, cellists, sanitary installations, and wall-mounted telephones. Vibrations are then sent straight to the structure [36]. The same strategy is used to prevent these sorts of sounds from traveling through a wall or floor, trying to keep the wall itself from vibrating as much as possible. However, this principle of sound insulation is handled in quite different ways for the two types of sound. The best technique to avoid structure-borne sound transmission is to absorb vibrations near the source. The majority of the vibrating energy will be absorbed by flexible mountings of sound-generating items, preventing it from continuing into the structure. It is worth noting that, in many circumstances, structure-borne sound can be controlled entirely with good design and detailing. In general, acoustic ratings are measures of sound transmission in building materials and assemblies. The two types of acoustic ratings discussed here are sound transmission loss coefficients (TLC) and sound transmission class (STC). The TLC of a material in a specified frequency band is measured in decibels, dB, and is ten times the common logarithm of the reciprocal of the sound transmission coefficient [37,38]. STC is the measure, in decibels, of how much airborne sound a floor or wall assembly blocks. STC ratings only measure sound blocked in the 125 to 4000 Hz range, which corresponds to normal and amplified speech. A wall assembly with an STC rating of 50 can reduce 110 dB of airborne sound generated on one side of the wall to 60 dB of airborne sound on the other. This is the equivalent of reducing the noise level of a rock concert to the level of normal speech [26,39].

**Figure 13:**
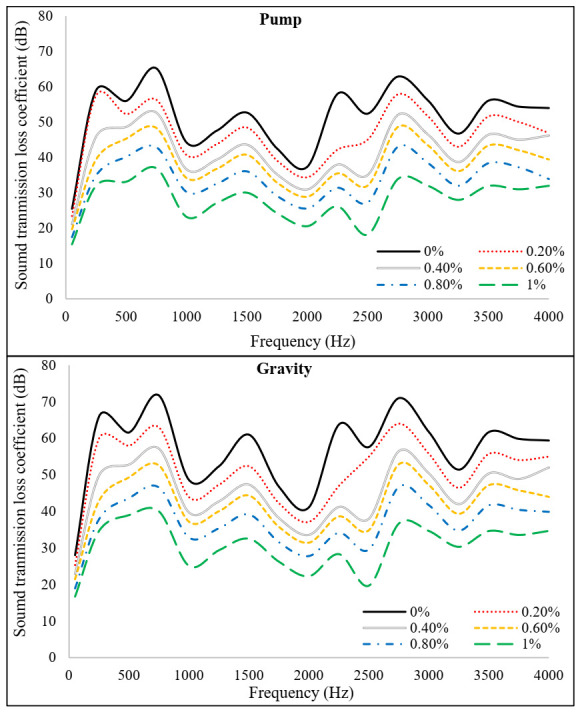
Sound transmission loss coefficient of PAFRC mixtures.

**Figure 14:**
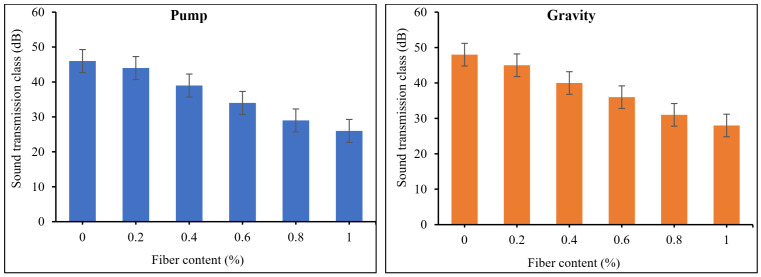
Sound transmission class PAFRC mixtures.

**Figure 15:**
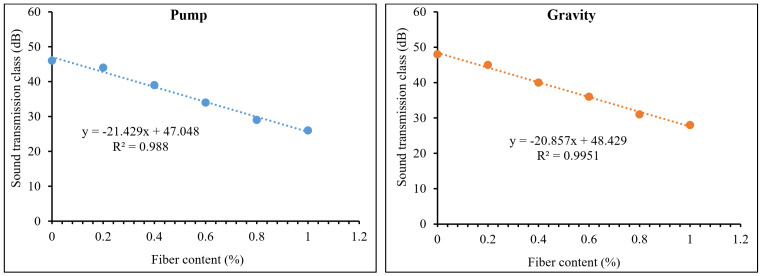
Correlations between the sound transmission class and fiber dosages of PAFRC mixtures.

Figure 13 and Figure 14 exhibit the recorded transmission loss coefficients (TLC) and sound transmission class (STC) results for PAFRC mixtures. It was observed that mixes with PP fibers had lower TLC and STC values than plain concrete. At a frequency of 2000 Hz, both gravity and pumping method mixtures have a TLC range of 20–38 dB, which is lower than that obtained for plain mixes. It means that PP fibers absorb and decrease sound energy more effectively than ordinary concrete elements. The function of PP fibers in the matrix is to act as sound traps, preventing the sound of landing on the surface from penetrating the material. The fibers’ linked behavior led to lower STC values than plain mixes. The TLC dropped as the volume of voids in the PAFRC mixtures increased due to increased fiber content because the sound may easily pass through the air void present inside the matrix. In addition, the sound transmission class and fiber volume fractions were shown to be correlated. Data analysis and linear regression were used to discover these correlations. Figure 15 shows the empirical equations derived between the sound transmission class and fiber content for both gravity and pumping method PAFRC specimens. According to the data, the fiber content and acoustic properties of the PAFRC mixes had a linear relationship, with coefficients of determination (R^2^) values greater than 0.98. According to the findings, increasing the fiber dosages improved the acoustic properties of the PAFRC mixes. Furthermore, the density of a material has a substantial impact on TLC [40,41,42]. As shown in Table 3, adding waste PP fibers with a density of 910 kg/m^3^ decreases the density of the concrete matrix. It can be observed that with the addition of fibers and increase in fiber dosages, the TLC and STC of the reinforced PAFRC mixture significantly reduced. In this regard, Holmes et al. [43] looked at the influence of waste crumb rubbers as a partial aggregate on the transmission loss and sound absorption of concrete and reported similar results. They found that as the crumb rubber content grew, the volume of pores and voids increased in the concrete matrix. Consequently, the sound was quickly absorbed, resulting in decreased TLC values of concrete.

## 4. Conclusions

The results of an experimental investigation into the mechanical and acoustic properties of PAFRC mixtures and how the combination of POFA and different dosages of PP fibers affect the performance of PAFRC are reported. Concerning the outcomes described in the current paper, the following conclusions could be drawn:

- The compressive strength of PAFRC mixes was marginally decreased when PP fibers were added, regardless of fiber dose. Due to the uniform circulation of grout under pressure, the pumping technique PAFRC specimens had greater strength values after one year of curing than the gravity technique PAFRC samples. Pozzolanic activity and hydration product development were both enhanced by the presence of POFA.
- The beneficial effect of PP fibers on improving the tensile strength was more considerable, likely owed to the bridging action and adequate bonding of fibers with the matrix. At one year of curing, the highest tensile strength of 5.2 MPa was recorded for the pumping method PAFRC mixture containing 0.6%, about 34% greater than that of 3.9 MPa noted for the plan mix.
- The attained UPV values for both grouting methods of PAFRC mixtures were recorded higher than 4400 m/s, indicating good quality concrete mixtures. Besides, the UPV and compressive strength values of PAFRC mixtures were correlated and demonstrated a linear relationship.
- The PAFRC mixes containing 0.2–1% PP fibers showed satisfactory sound absorption coefficients of up to 63% in the medium and high-frequency regions, which is comparatively higher than the 15% noted for the plain mixes. The average sound absorption and noise reduction of pumping group mixes were better than that of gravity method PAFRC mixes.
- The sound transmission class and sound transmission loss coefficient for PAFRC mixtures comprising PP fibers decreased associated with plain PAC mix. The function of fibers in the concrete matrix is to act as sound traps, and therefore, trap and absorb the sound that entered inside the matrix.

## Figures and Tables

**Table 1:** Properties of OPC and POFA.

Compositions	OPC (%)	POFA (%)
SiO_2_	20.4	62.6
Al_2_O_3_	5.2	4.6
Fe_2_O_3_	4.2	8.1
CaO	62.4	5.7
MgO	1.56	3.5
K_2_O	0.005	9.04
SO_3_	2.12	1.15
LOI	2.35	6.24
Physical properties		
Specific gravity	3.15	2.42
Blaine fineness (cm^2^/g)	3990	4930
Soundness (mm)	1.0	2.0

**Table 2:** Properties of polypropylene fibers used in this study.

Type of Fiber	Length (mm)	Diameter (µm)	Density (kg/m^3^)	Tensile Strength (MPa)	Melting Point (^o^C)
Multi-filament polypropylene	30 ± 2	45	910	400	170

**Table 3:** PAFRC mix proportions.

Mix	Water (kg/m^3^)	Cement (kg/m^3^)	POFA (kg/m^3^)	Fine Agg (kg/m^3^)	Coarse Agg (kg/m^3^)	V_f_ (%)	Dry Density (kg/m^3^)
P-0	180	293	97	550	1315	0.0	2430.0
P-1	180	293	97	550	1315	0.2	2308.5
P-2	180	293	97	550	1315	0.4	2223.5
P-3	180	293	97	550	1315	0.6	2162.7
P-4	180	293	97	550	1315	0.8	2133.5
P-5	180	293	97	550	1315	1.0	2114.1
G-0	180	293	97	550	1315	0.0	2430.0
G-1	180	293	97	550	1315	0.2	2279.3
G-2	180	293	97	550	1315	0.4	2213.7
G-3	180	293	97	550	1315	0.6	2138.4
G-4	180	293	97	550	1315	0.8	2126.3
G-5	180	293	97	550	1315	1.0	2109.2

## Data Availability

The data presented in this study are available on request from the corresponding author. The data are not publicly available due to size of a research.
